# Computed tomography-measured trabecular attenuation at first lumbar vertebra as a surrogate marker of bone mineral density and osteoporosis in patients undergoing hemodialysis

**DOI:** 10.1080/0886022X.2026.2671455

**Published:** 2026-05-20

**Authors:** Takahiro Yajima, Ai Kurisawa, Maiko Arao

**Affiliations:** Department of Nephrology, Matsunami General Hospital, Gifu, Japan

**Keywords:** Bone mineral density, computed tomography, CT-attenuation, hemodialysis, osteoporosis

## Abstract

The risk of fractures and osteoporosis is higher in patients on hemodialysis than in the general population. We examined the association between computed tomography (CT)-measured trabecular attenuation at the first lumbar vertebra (L1 CT-attenuation), bone mineral density (BMD), and osteoporosis in patients on hemodialysis. We retrospectively included Japanese patients on hemodialysis who underwent non-enhanced abdominal CT and dual-energy X-ray absorptiometry (DEXA) analyses as part of a medical checkup between April 2019 and March 2021. L1 CT-attenuation (Hounsfield units, HU) and BMD of the femoral neck and lumbar spine were measured. We analyzed associations between L1 CT-attenuation and BMD and examined its diagnostic accuracy and odds ratio for detecting osteoporosis, defined as a DEXA T-score of < −2.5 at either site. A total of 119 patients (mean age: 67.2 years; men; 63.0%; body mass index [BMI]: 22.4 ± 3.9 kg/m^2^) were included, of whom 49 had osteoporosis. L1 CT-attenuation was independently linked to BMD at the femoral neck and lumbar spine. The optimal cutoff derived from receiver operating characteristic curve analysis was 109.3 HU. Thresholds of ≤71.3 HU and ≥151.1 HU provided 90% specificity and 90% sensitivity for distinguishing patients with and without osteoporosis. The age-, sex-, and BMI-adjusted odds ratio of L1 CT-attenuation (per 10 HU) for diagnosing osteoporosis was 0.73. L1 CT-attenuation was independently associated with BMD and may be utilized for osteoporosis screening in Japanese patients on hemodialysis. Opportunistic abdominal CT may aid early detection and intervention for osteoporosis in these patients.

## Introduction

Osteoporosis is a bone disorder characterized by decreased bone strength, which adversely affects bone quantity and quality and increasing fracture risk. The Kidney Disease: Improving Global Outcomes (KDIGO) Controversies Conference introduced chronic kidney disease (CKD)-associated osteoporosis as a new conceptual framework within CKD–mineral and bone disorders [1]. The occurrence of fractures is higher in patients with CKD than in the general population due to both CKD-specific and traditional risk factors [1,2]. Traditional risk factors include age and menopause, whereas CKD-specific risk factors include disturbances in mineral metabolism, uremic toxins, immune (systemic inflammation), endocrine (hyperparathyroidism and vitamin D deficiency), and neurohormonal disturbances, as well as a long dialysis duration [1,2]. Moreover, patients with CKD often have secondary risk factors for osteoporosis, such as hypogonadism, glucocorticoid use, diabetes mellitus, low body mass index (BMI), and malnutrition [1–3]. Because bone fractures are associated with a high risk of cardiovascular events and mortality [4–7], therefore, preventing osteoporosis is crucial in patients undergoing hemodialysis.

In patients with CKD, a history of fragility fractures or T-scores < −2.5 must be considered indicative of osteoporosis, regardless of disturbances in mineral metabolism [8]. Bone mineral density (BMD) is typically measured to assist in diagnosing and monitoring osteoporosis using dual-energy X-ray absorptiometry (DEXA). A Japanese cohort study revealed the association between a decreased BMD and an increased fracture risk in patients undergoing hemodialysis [9]. Thus, guidelines for CKD–mineral and bone disorders recommend BMD assessment in patients undergoing hemodialysis [10]. However, not all patients undergo DEXA. Recently, opportunistic assessment of trabecular attenuation values in vertebrae using routine abdominal computed tomography (CT) has gained attention as an increasingly valuable method for identifying high-risk patients for osteoporosis [11–26]. Among these approaches, average CT-attenuation values (in Hounsfield units, HU) at the first lumbar vertebral level (L1 CT-attenuation) has emerged as a reliable and clinically practical surrogate for BMD. Trabecular attenuation measured at the L1 level is as accurate as, or more accurate than, measurements at other levels for predicting osteoporosis [11]. Additionally, the L1 level is easy to identify and is included in standard chest and abdominal CT scans [11]. Thus, L1-level measurements have been widely used in studies of prior opportunistic osteoporosis screening [11–26]. Pickhardt et al. performed a cross-sectional analysis of patients who underwent abdominal CT and DEXA within 6 months and first proposed practical HU thresholds: < 110 HU strongly indicates osteoporosis, whereas > 160 HU indicates that osteoporosis is unlikely [11]. Thereafter, various cutoffs have been reported in different ethnic and clinical settings [15,22,24–26]. However, to our knowledge, the relationship between L1 CT-attenuation, BMD, and osteoporosis prediction has not been examined in patients on hemodialysis. Thus, the present study aimed to examine the association among L1 CT-attenuation, BMD, and osteoporosis in these patients.

## Methods

### Participants and inclusion and exclusion criteria

This study retrospectively enrolled adult patients aged ≥18 years who had been receiving maintenance hemodialysis for ≥6 months and underwent plain abdominal CT and DEXA scans of the lumbar spine and femoral neck within the same week from April 2019 and March 2021 at Matsunami General Hospital, Kasamatsu, Gifu. At our institution, CT and DEXA scans are included in the annual checkup. Patients with bilateral femoral neck fractures and bedridden patients were excluded. We also excluded patients with symptomatic lumbar compression fractures at the L1 level or those who had undergone surgery, as L1 CT-attenuation or lumbar spine BMD could not be measured appropriately in these cases. This study was conducted in accordance with the Declaration of Helsinki and was approved by the institutional review board of Matsunami General Hospital (‘Ethics Committee of Matsunami General Hospital Medical’; approval number: No. 643). The informed consent requirement was waived because the present retrospective study used chart reviews of previously performed CT and DEXA examinations.

### Collection of clinical data

We retrieved all patient information from medical charts, including age, sex, BMI, smoking and alcohol consumption (self-reported current alcohol use), underlying kidney disease (nephrosclerosis, chronic glomerulonephritis, diabetic kidney disease, and others), history of diabetes mellitus, duration of hemodialysis, and medications, including a phosphorus binder (especially calcium-containing), oral or parenteral vitamin D, oral or parenteral calcium-sensing receptor agonists, and osteoporosis therapies (bisphosphonate, romosozumab, and selective estrogen receptor modulator). A blood test was performed in the supine position before the initiation of hemodialysis on Mondays or Tuesdays. Laboratory data and findings of CT and DEXA performed within 1 month were used for data analyses.

### Measurement of L1 CT-attenuation values

**Figure 1.**
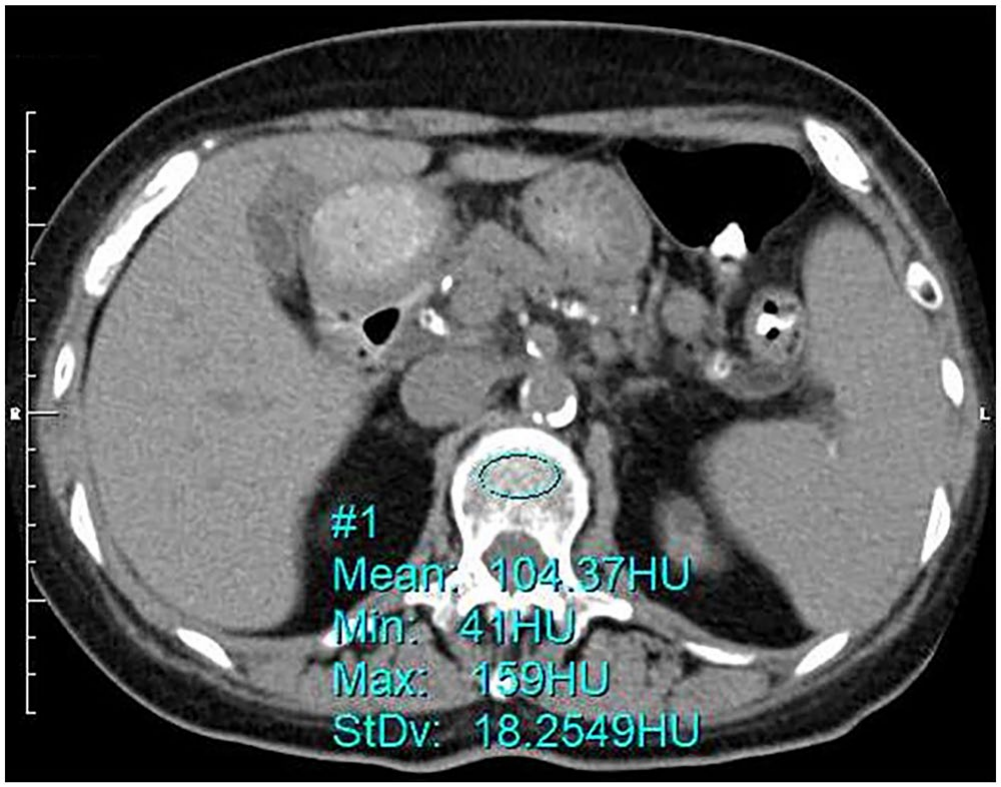
An example of measuring L1 CT-attenuation value. A single axial computed tomography (CT) slice of the first lumbar (L1) vertebral body was selected at the pedicular level. The region of interest (ROI) tool was used. The ROI was set to the maximum possible elliptical diameter without including the vertebral cortex or the basivertebral venous plexus. The average CT value within the ROI was defined as L1 CT-attenuation. In this case, the L1 CT-attenuation was 104.37 HU.

Non-enhanced abdominal CT was performed using a multidetector CT scanner (LightSpeed Series; GE HealthCare, Chicago, IL, USA). All CT scans were performed at 120 kVp with a 5 mm slice thickness and a 512 × 512 matrix. The in-plane pixel spacing, derived from the DICOM metadata, ranged from approximately 0.51 to 0.97 mm, depending on the field of view. A single axial computed tomography (CT) slice through the center of the L1 vertebral body was selected at the pedicular level. L1 was defined as the first non-rib-bearing vertebra. HU measurements were retrospectively assessed using a picture archiving and communication system (XTREK View; J-MAC System, Inc., Sapporo, Japan) with a region-of-interest (ROI) tool. For each patient, the ROI was manually placed over the trabecular bone of L1, with the maximum possible elliptical diameter, avoiding the vertebral cortex and the posterior venous plexus, as previously reported (Figure 1) [11,19,26]. The mean CT value (HU) within the ROI was defined as the L1 CT-attenuation. An example of L1 CT-attenuation measurement is shown in Figure 1. Two examiners, MA and TY, measured L1 CT-attenuation. They could vary the selection of an axial slice at the L1 level and the placement and size of the ROI within the trabecular bone. Both examiners were masked to patients’ clinical information, including DXA results and laboratory data. Each observer was also masked to the other’s measurements.

### BMD measurement using DEXA

BMDs measurements at the proximal femoral neck and lumbar vertebrae were assessed using standard techniques on a DEXA scanner with Smart BMD software (SONIALVISION G4; Shimadzu Corporation, Kyoto, Japan). BMD is expressed in g/cm^2^. Osteoporosis was defined as a low DEXA T-score of < −2.5 at either the femoral neck or lumbar spine, according to the WHO criteria [27,28].

### Statistical analyses

Non-normally distributed variables are described as medians and interquartile ranges and normally distributed variables by means and standard deviations. Intra- and inter-rater reproducibility of L1 CT-attenuation values were evaluated using the intraclass correlation coefficient (ICC) and 95% confidence interval (CI). ICC >0.9 was considered excellent for reproducibility and clinical acceptability. The agreement was further evaluated using a Bland–Altman analysis. Continuous variables were compared using the t-test or the Wilcoxon signed-rank test and categorical variables using the chi-squared test between patients with and without osteoporosis. The correlations among L1 CT-attenuation and T-scores of the femoral neck and lumbar spine were shown with scatter plots. Correlation between L1 CT-attenuation and femoral neck or lumbar spine BMD was assessed with Pearson’s correlation coefficient. Before applying linear models, the linearity between L1 CT-attenuation and BMD was visually assessed using scatter plots. Univariable regression analysis was performed to evaluate associations between baseline variables and L1 CT-attenuation. Thereafter, multivariable regression analysis was performed with a model that included all significant variables from the univariable regression analysis. BMDs at the femoral neck and at the lumbar spine were analyzed in separate models to avoid multicollinearity arising from their strong correlation. We assessed multicollinearity using variance inflation factors (VIFs). The optimal cutoff value of L1 CT-attenuation for diagnosing osteoporosis was determined using receiver operating characteristic (ROC) curve analysis with the Youden index. In addition, we determined the L1 CT-attenuation thresholds that demonstrated high sensitivity (≥ 90%) and specificity (≥ 90%) for diagnosing osteoporosis. To account for potential non-linear relationships and to further assess the robustness of the association, logistic regression analyses were performed with osteoporosis (defined as a T-score < −2.5) as the outcome. Non-linearity was evaluated using restricted cubic splines with three knots. Multivariable models were adjusted for clinically relevant covariates, including age, sex, and BMI.

Furthermore, unadjusted and sex–age–BMI-adjusted odds ratios (ORs) and 95% CIs for L1 CT-attenuation values were evaluated to assess their association with osteoporosis. Most variables were available for all participants; however, 25(OH)D levels were measured in a subset of patients (*n* = 90) because of the retrospective design of the present study. Analyses involving this variable were conducted using the available data. If minor missing data were present, a complete case analysis was performed. We used SPSS Statistics for Windows, version 31 (IBM Corp., Armonk, NY, USA) for statistical analyses and JMP^®^, version 19 (JMP Statistical Discovery LLC, Cary, NC, USA). A p-value < 0.05 was considered significant.

## Results

### Background characteristics of study participants

We screened 128 Japanese patients receiving maintenance hemodialysis and excluded two patients with bilateral fractures of the femoral neck (bedridden status) and 7 patients with symptomatic vertebral fractures. We included 119 patients (mean age: 67.2 years; men: 63%; BMI: 22.4 kg/m^2^), of whom 49 patients were diagnosed with osteoporosis. Baseline characteristics are summarized in Table 1.

**Table 1.** Background characteristics of the study participants.

Variables	All patients *(N* = 119)	Non-osteoporosis (*n* = 70)	Osteoporosis (*n* = 49)	p value
Age (year)	67.2 ± 12.7	64.7 ± 13.0	70.9 ± 11.3	0.0076
Male (%)	63.0	82.9	34.7	<0.0001
Body mass index (kg/m^2^)	22.4 ± 3.9	23.8 ± 3.8	20.5 ± 3.2	<0.0001
Smoking (%)	22.7	22.9	22.4	0.96
Alcohol consumption (%)	28.6	32.9	22.4	0.21
Underlying kidney disease				0.74
Diabetic nephropathy	41.2	44.3	36.7	
Chronic glomerulonephritis	26.1	25.7	26.5	
Nephrosclerosis	23.5	22.9	24.5	
Others	9.2	7.1	12.2	
Diabetes mellitus (%)	43.6	48.6	36.7	0.20
Hemodialysis duration (years)	3.3 (1.7–6.4)	3.5 (2.0–6.2)	3.3 (1.3–6.8)	0.57
Medications (%)				
Calcium-containing phosphorous binder (%)	50.4	60.0	36.7	0.012
Vitamin D (%)	58.8	57.1	61.3	0.90
Oral (%)	7.6	7.1	8.2	
Parenteral (%)	51.2	50.0	53.1	
Calcium-sensing receptor agonists (%)	13.4	10.0	18.4	0.24
Oral (%)	7.6	7.1	8.2	
Parenteral (%)	5.9	2.9	10.2	
Therapy for osteoporosis (%)	12.6	0	30.6	<0.0001
Bisphosphonate (%)	4.2	0	10.2	
Romosozumab (%)	7.6	0	18.4	
Selective estrogen receptor modulator (%)	0.8	0	2.0	
Serum albumin (g/dL)	3.4 ± 0.3	3.4 ± 0.3	3.3 ± 0.3	0.14
Total cholesterol (mg/dL)	145 ± 38	140 ± 42	153 ± 32	0.063
Serum creatinine (mg/dL)	9.4 ± 3.2	10.0 ± 3.5	8.7 ± 2.4	0.029
Calcium (mg/dL)	8.6 ± 0.6	8.6 ± 0.6	8.5 ± 0.7	0.50
Phosphorus (mg/dL)	4.6 ± 1.1	4.5 ± 1.2	4.7 ± 1.1	0.46
Intact parathyroid hormone (pg/mL)	135 (63–207)	134 (63–191)	143 (67–243)	0.23
25(OH)D (ng/mL) (*n* = 90)	11.9 ± 5.6 (*n* = 90)	12.2 ± 5.3 (*n* = 56)	11.4 ± 6.1 (n = 34)	0.52
C-reactive protein (mg/dL)	0.10 (0.03–0.28)	0.12 (0.03–0.36)	0.08 (0.04–0.19)	0.35
Bone mineral density at the femoral neck (g/cm^2^)	0.70 ± 0.11	0.78 ± 0.08	0.60 ± 0.06	
Femoral neck T-score	−2.11 ± 1.03	−1.46 ± 0.73	−3.04 ± 0.58	
Bone mineral density at the lumbar spine (g/cm^2^)	1.02 ± 0.18	1.11 ± 0.13	0.88 ± 0.15	
Lumber spine T-score	−1.19 ± 1.24	−0.54 ± 0.93	−2.13 ± 1.00	
CT-measured trabecular attenuation at the L1 vertebral level (Hounsfield units)	110.3 ± 41.0	122.3 ± 40.2	93.1 ± 35.9	<0.0001

CT, computed tomography.

### Reproducibility of L1 CT-attenuation measurements

**Figure 2.**
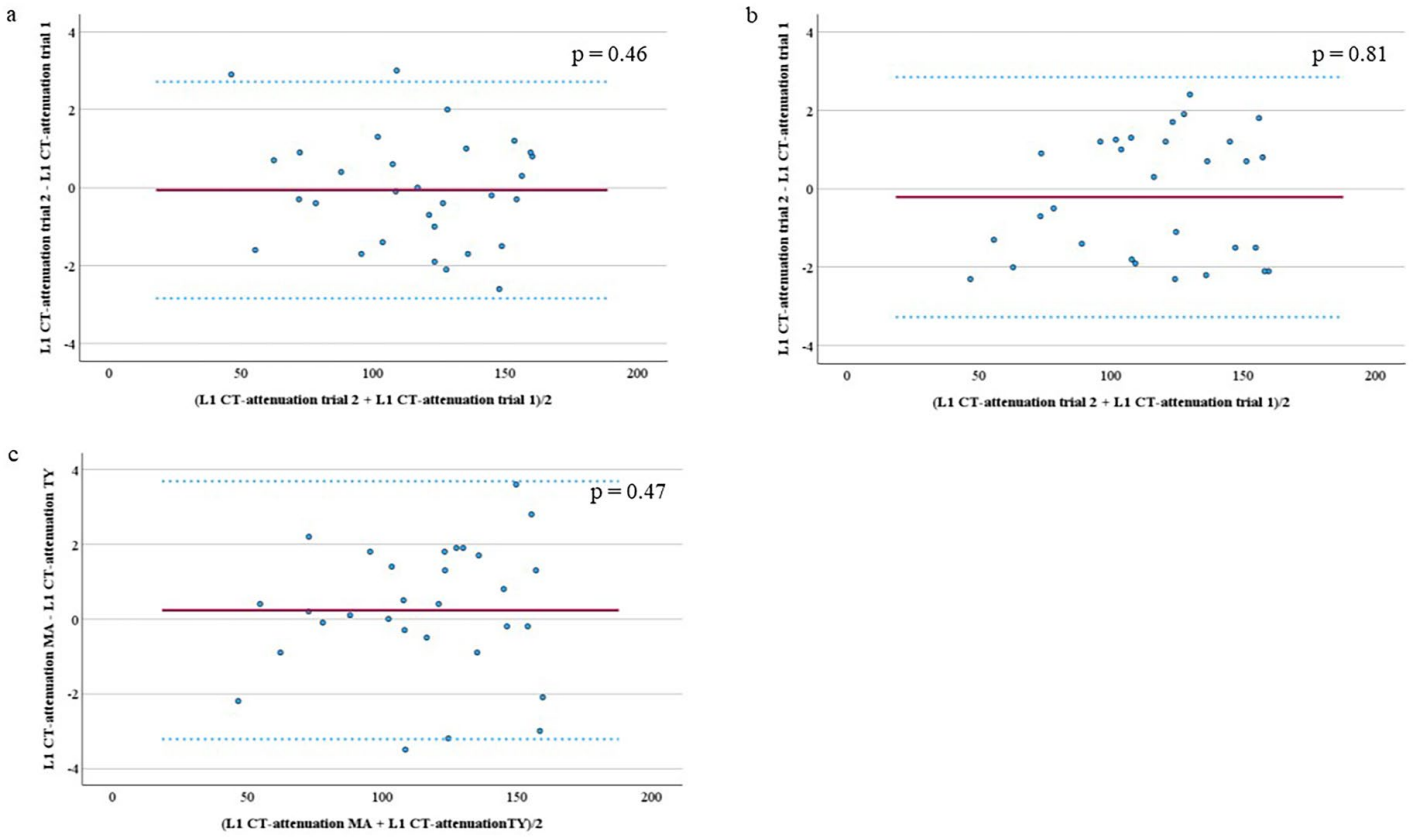
Bland–Altman analysis to assess agreement of L1 computed tomography (CT)-attenuation values. Two investigators (MA and TY) measured L1 CT-attenuation values and described the differences and 95% confidence intervals for the plots. Intra-rater agreement of MA (a) and that of TY (b), and inter-rater agreement between MA and TY (c) were shown.

Intra- and inter-rater reproducibility and agreement of L1 CT-attenuation measurements were assessed in 30 randomly selected patients using ICCs and Bland–Altman analysis. Intra-rater ICCs were 0.999 (95% CI: 0.998–0.999) for MA and 0.999 (95% CI: 0.998–1) for TY, and the inter-rater (MA and TY) ICC was 0.999 (95% CI: 0.997–0.999). Bland–Altman analysis demonstrated good intra-rater and inter-rater agreement (Figure 2).

### Association between L1 CT-attenuation and bone mineral density of the femoral neck and lumbar spine

**Figure 3.**
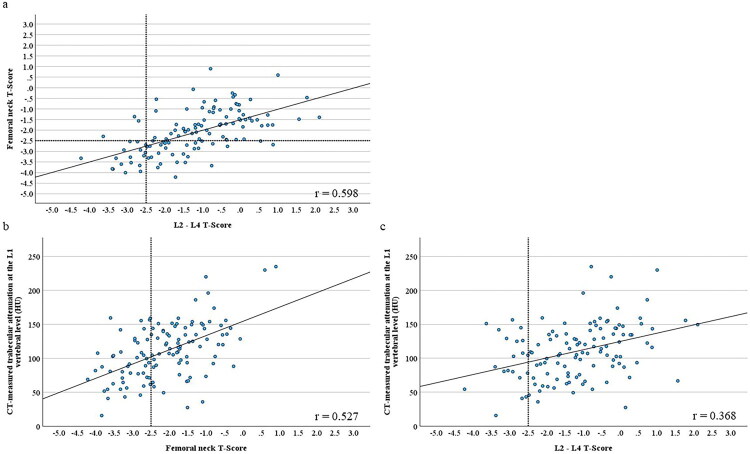
Correlation between CT-measured trabecular attenuation at the L1 vertebral level and T-scores of the femoral neck and lumbar spine. The correlation of T-scores between the femoral neck and the lumbar spine (a), and the correlation between CT-measured trabecular attenuation at the L1 vertebral level and T-scores of either the femoral neck (b) or lumbar spine (c) were shown. The horizontal and vertical reference lines indicate osteoporosis thresholds according to DXA criteria (T-score < −2.5).

The positive correlation of T-scores between the femoral neck and the lumbar spine (Figure 3a), and the positive correlation between CT-measured trabecular attenuation at the L1 vertebral level and T-scores of either the femoral neck (Figure 3b) or lumbar spine (Figure 3c) were shown. Femoral neck and lumbar spine BMDs were significantly correlated (*r* = 0.598; *p* < 0.0001). Moreover, L1 CT-attenuation was significantly correlated with the femoral neck (*r* = 0.527; *p* < 0.0001) and lumbar spine (*r* = 0.368; *p* < 0.0001) BMDs (Table 2). L1 CT-attenuation was significantly correlated with age, diabetes mellitus, use of calcium-containing phosphorus binder or therapy for osteoporosis, and serum creatinine levels in the univariable regression analysis (Table 2). In addition, L1 CT-attenuation was independently associated with the femoral neck (β = 0.319; *p* = 0.0003) and lumbar spine (β = 0.265; *p* = 0.0006) BMDs in the multivariable regression analysis (Table 3).

**Table 2.** Associations between CT-measured trabecular attenuation at the L1 vertebral level and baseline variables.

Variables	Univariable (r)	p value
*Age*	−0.579	<0.0001
Sex (male)	0.035	0.71
Body mass index	0.170	0.065
Smoking	0.080	0.39
Alcohol	0.103	0.26
*Diabetes mellitus*	0.238	0.0092
Hemodialysis vintage	0.146	0.11
Medications		
*Calcium-containing phosphorus binder*	0.195	0.033
Vitamin D	0.097	0.29
Calcium-sensing receptor agonists	−0.148	0.11
*Therapy for osteoporosis*	−0.194	0.034
Serum albumin	0.148	0.11
Total cholesterol	−0.064	0.49
*Serum creatinine*	0.236	0.0097
Calcium	0.138	0.14
Phosphorus	0.057	0.54
Intact parathyroid hormone	−0.102	0.27
25(OH)D (*n* = 90)	−0.147	0.17
C-reactive protein	−0.054	0.56
*Bone mineral density at the femoral neck*	0.527	<0.0001
*Femoral neck T-score*	0.527	<0.0001
*Bone mineral density at the lumbar spine*	0.368	<0.0001
*Lumber spine T-score*	0.368	<0.0001

CT, Computed tomography.

**Table 3.** Associations between CT-measured trabecular attenuation at the L1 vertebral level and BMD measured at the femoral neck and lumbar spine.

Variables	Multivariable model including femoral neck BMD (β)	p value	VIF	Multivariable model including lumbar spine BMD (β)	p value	VIF
Age	−0.486	<0.0001	1.66	−0.596	<0.0001	1.42
Diabetes mellitus	0.152	0.034	1.06	0.128	0.079	1.11
Calcium-containing phosphorus binder	0.024	0.74	1.13	0.021	0.78	1.13
Therapy for osteoporosis	−0.022	0.78	1.37	−0.094	0.20	1.14
Serum creatinine	0.078	0.35	1.51	0.107	0.21	1.49
*BMD at the femoral neck*	0.319	0.0003	1.57	–	–	
*BMD at the lumbar spine*	–	–		0.265	0.0006	1.18

CT, computed tomography; BMD, bone mineral density; VIF, variation inflation factor.

### L1 CT-attenuation cutoffs for diagnosing osteoporosis

**Figure 4.**
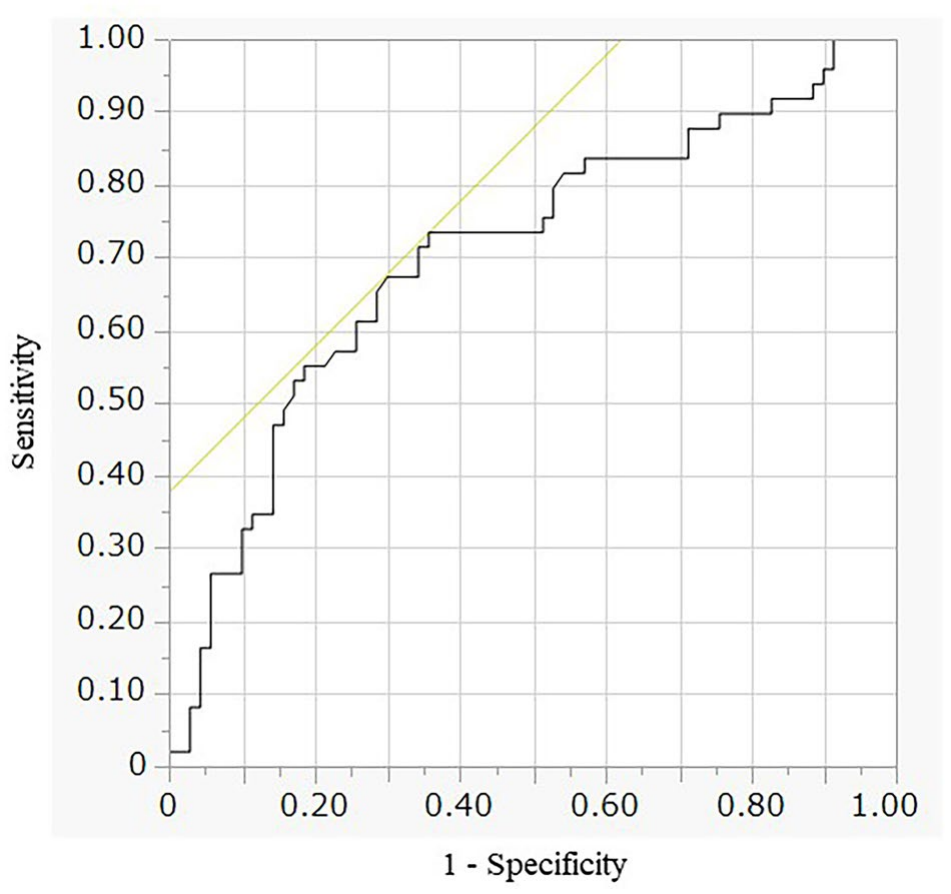
Receiver operating characteristic (ROC) analysis of CT-measured trabecular attenuation at the L1 vertebral level for osteoporosis diagnosis. Osteoporosis was defined as a low DEXA-measured T-score of < −2.5. T-scores for the femoral neck and lumbar spine were evaluated, and the lower value among these scores was used.

The ROC-derived optimal cutoff for L1 CT-attenuation was 109.3 HU (area under the curve [AUC], 0.705; sensitivity, 0.735; specificity, 0.643; *p* < 0.0001) (Figure 4). An L1 CT-attenuation threshold of 71.3 HU achieved ≥90% specificity, whereas a threshold of 151.1 HU achieved ≥90% sensitivity for diagnosing osteoporosis.

### Association between L1 CT-attenuation and osteoporosis

**Figure 5.**
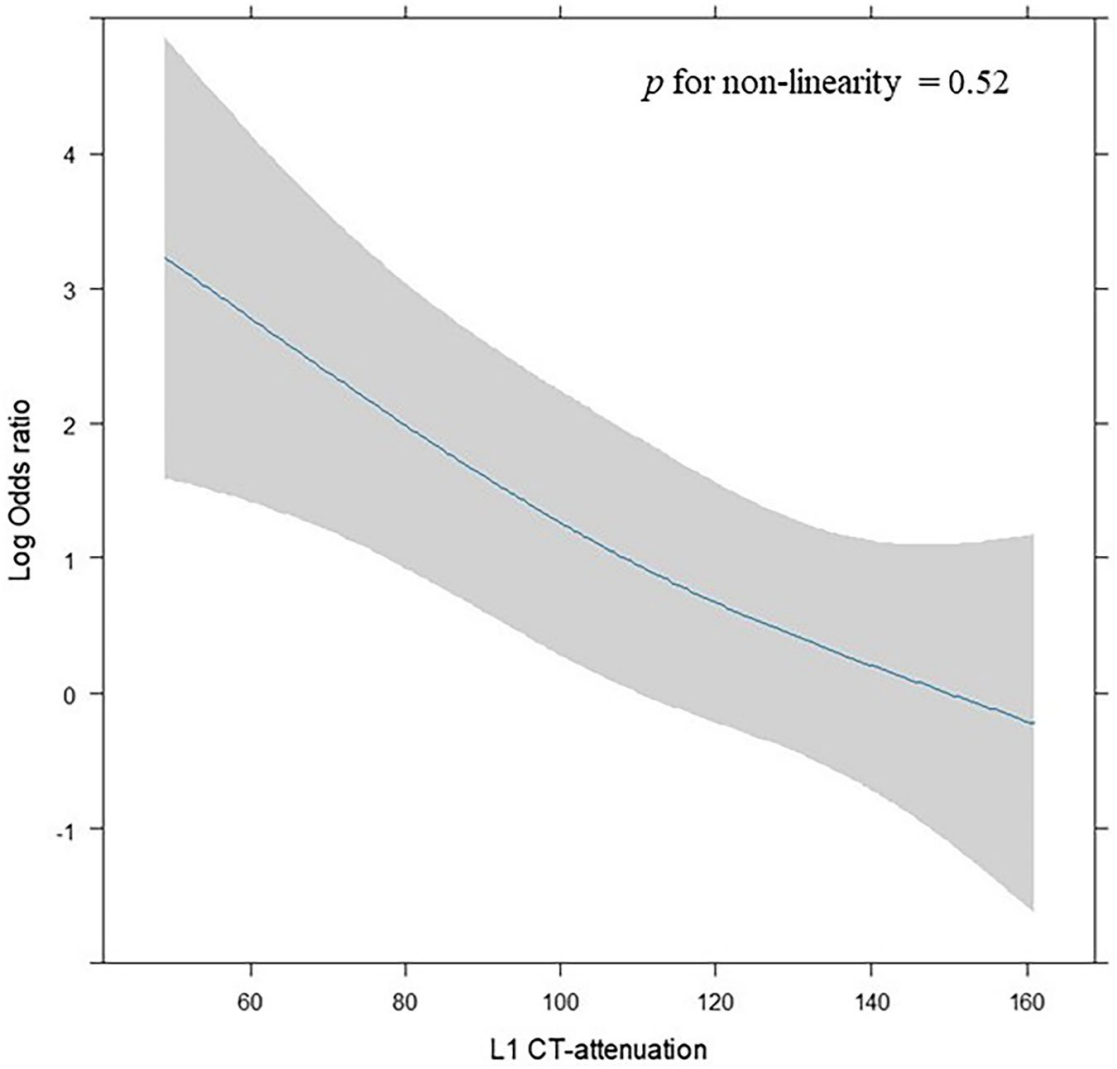
Association between L1 CT-attenuation and osteoporosis using restricted cubic spline analysis. The curve shows the adjusted log odds ratio for osteoporosis by L1 CT-attenuation, with 95% confidence intervals shown as gray-shaded areas. The model was adjusted for age, sex, and body mass index. No significant non-linearity was observed (p for non-linearity = 0.52), supporting a linear relationship.

In logistic regression analyses with restricted cubic splines, L1 CT-attenuation was significantly associated with osteoporosis both before and after adjusting for covariates (*p* = 0.0012 and *p* = 0.0041, respectively). There was no evidence of non-linearity in the association (p for non-linearity = 0.82 in the unadjusted model and *p* = 0.52 in the adjusted model), indicating that the relationship between L1 CT-attenuation and osteoporosis can be adequately described as linear (Figure 5).

### Unadjusted and adjusted ORs of L1 CT-attenuation for diagnosing osteoporosis

The unadjusted OR for diagnosing osteoporosis per 10-HU-increase in L1 CT-attenuation was 0.817 (95% CI, 0.722–0.904; *p* < 0.0001). Additionally, the sex–age–BMI-adjusted OR for diagnosing osteoporosis per 10-HU-increase in L1 CT-attenuation was 0.73 (95% CI, 0.592–0.868; *p* = 0.0003).

## Discussion

To the best of our knowledge, this is the first study to examine the association among L1 CT-attenuation, BMD, and osteoporosis in Japanese patients on hemodialysis. L1 CT-attenuation was independently associated with the femoral neck and lumbar vertebral BMD. The optimal L1 CT-attenuation cutoff for diagnosing osteoporosis was 109.3 HU, which demonstrated moderate diagnostic accuracy (AUC: 0.705). Moreover, thresholds of ≥151.1 HU exhibited over 90% sensitivity, whereas a threshold of 71.3 HU or lower exhibited over 90% specificity for distinguishing between patients with and without osteoporosis. After adjusting for age, sex, and BMI, a low L1 CT-attenuation value was independently associated with osteoporosis. Therefore, CT screening may facilitate the early detection and intervention for osteoporosis in this population.

In this study, we measured L1 CT-attenuation using an abdominal CT image for kidney cancer screening. Two masked investigators evaluated L1 CT-attenuation in 30 randomly selected patients, demonstrating excellent reproducibility and agreement of L1 CT-attenuation measurements, as previously reported in healthy individuals [29]; this finding indicates that L1 CT-attenuation may be a clinically acceptable biomarker in patients on hemodialysis.

We examined the association between L1 CT-attenuation and BMD of the femoral neck and lumbar spine. Previous studies have reported stronger correlations of L1 CT-attenuation with lumbar spine BMD than with femoral neck BMD (*r* = 0.552 vs. 0.349 and 0.726 vs. 0.503, respectively) [18,30]. Conversely, in this study, the correlation between L1 CT-attenuation and femoral neck BMD (*r* = 0.527) was stronger than that between L1 CT-attenuation and lumbar spine BMD (*r* = 0.368). This may reflect that DEXA findings incorporate posterior spinal elements, which can lead to inaccuracies in cases of previous lumbar surgery, scoliosis, or severe spinal degeneration [31,32]. In addition, the lumbar spine BMD may be overestimated in patients with CKD due to vascular calcification [33,34]. Although we excluded patients with a history of L1 vertebral fracture or lumbar surgery, the BMD measurement of the lumbar vertebra might have been less accurate than that of the femoral neck owing to lumbar degeneration or vascular calcification. Moreover, the number of patients with osteoporosis with a T-score of femoral neck < − 2.5 was higher than the number of patients with a T-score of lumbar spine < − 2.5 (*N* = 46 vs. *N* = 20, respectively; details not shown). Because L1 CT-attenuation was derived from trabecular bone regions, the association between vertebral cortical bone and BMD could not be assessed in the present study. In CKD-mineral and bone disorders, trabecular bone is closely linked to changes in bone turnover, whereas cortical bone abnormality contributes to bone fragility and fracture risk. Notably, cortical bone involvement is prominent, with increased cortical porosity representing a major skeletal abnormality in secondary hyperparathyroidism [35]. Thus, this pathophysiology might partly explain why L1 CT-attenuation correlated more strongly with femoral neck BMD than with lumbar spine BMD. However, whether the lumbar spine’s cortical bone is similarly affected by secondary hyperparathyroidism as the femoral neck’s cortical bone remains unknown.

Importantly, L1 CT-attenuation was independently associated with DEXA-measured BMDs of the femoral neck and lumbar vertebrae after adjusting for age, diabetes mellitus, use of calcium-containing phosphorus binders or osteoporosis therapy, and creatinine levels. These findings indicate that L1 CT-attenuation is a useful marker of BMD in patients undergoing hemodialysis. However, we have to acknowledge that some variables (i.e., age, BMD, and osteoporosis therapy) may not be fully independent in the multivariable regression models. We assessed multicollinearity using VIFs and observed no significant collinearity among the included variables. Age is a well-known determinant of both BMD and L1 CT-attenuation values; therefore, age was included in the model to adjust for its confounding effect on the association between L1 CT-attenuation and BMD. Regarding osteoporosis therapy, we acknowledge that it is not independent of BMD, as it is typically initiated in patients with decreased BMD. For this reason, osteoporosis therapy was included as a covariate to account for its potential modifying effect rather than assuming independence. To assess this, we added a sensitivity analysis assessing the associations between L1 CT-attenuation and BMDs at the femoral neck and lumbar spine in patients who did not receive osteoporosis therapy (*N* = 104). Results were similar to those in the primary analyses: L1 CT-attenuation was independently associated with BMD at the femoral neck (β = 0.287, *p* = 0.0006) and with BMD at the lumbar spine (β = 0.254, *p* = 0.0012) (details not shown in the manuscript). Moreover, L1 CT-attenuation was independently associated with diabetes mellitus in the model including femoral neck BMD, but not in the model including lumbar spine BMD. The lumbar spine is predominantly trabecular, whereas the femoral neck contains a relatively higher proportion of cortical bone in addition to trabecular bone. Diabetes mellitus affects bone vulnerability through site-specific mechanisms with an increased cortical porosity in cortical bone rather than deterioration of trabecular microarchitecture in trabecular bone [36]. These differences in bone composition may have contributed to the observed discrepancy between models. In addition, given the relatively small sample size, the lack of significance in the model including lumbar spine BMD may be attributable to limited statistical power.

Thresholds for L1 CT-attenuation in diagnosing osteoporosis, including diagnostic performance and clinical interpretation, have been reported previously. Pickhardt et al. first reported an ROC-derived optimal threshold for L1 CT-attenuation of 135 HU with good diagnostic accuracy (AUC: 0.83) in a large USA-based cohort study [11]. Subsequently, many studies have shown that the optimal thresholds for osteoporosis diagnosis range from 95 to 180 HU, with moderate to good diagnostic accuracy (AUC 0.64–0.86) [12–23]. However, no such reports were found for the Japanese population; therefore, we could not compare our results with those of the general Japanese population. Nevertheless, within the Asian population, the optimal L1 CT-attenuation thresholds for osteoporosis diagnosis ranged from 95 to 136 HU, with AUCs of 0.74–0.86 [16–18,21,23]. In the present study, the optimal L1 CT-attenuation cutoff value for osteoporosis diagnosis was 109.3 HU (AUC: 0.705). This value was remarkably consistent with the thresholds reported in previous studies on Asian populations and had an acceptable discriminatory ability for osteoporosis diagnosis. Moreover, Pickhardt et al. proposed thresholds of < 110 HU for > 90% specificity and > 160 HU for > 90% sensitivity to discriminate between patients with and without osteoporosis [11]. Subsequently, several studies have reported different thresholds for osteoporosis diagnosis: 73–104 HU for > 90% specificity and 139–180 HU for > 90% sensitivity [15,22,24–26]. In this study, we identified clinically meaningful thresholds of ≤ 71.3 HU for > 90% specificity and ≥ 151.1 HU for > 90% sensitivity, which may be useful for screening and risk stratification of osteoporosis in practice.

Differences in cutoff values and diagnostic accuracies have been reported for various cohorts. These differences may reflect factors such as osteoporosis diagnosis, imaging modalities (including CT and DEXA), interval between CT and BMD measurements, and population characteristics (including race, sex, age, and background diseases). In this study, osteoporosis was diagnosed according to the WHO diagnostic criteria: a T-score of less than −2.5 at the femoral neck or lumbar spine was considered osteoporosis [27,28]. We performed CT and DEXA analyses, which are widely used techniques in Japan. Although in previous studies, the interval between CT and DEXA measurements varied (from 2 weeks to 2 years) [37], we performed CT and DEXA within the same week because they were part of a routine medical checkup for cancer screening at our hospital. Non-enhanced CT was performed at 120 kV to screen for renal cancer. CT attenuation is influenced by the kilovoltage, and these existing diagnostic thresholds are typically based on CT scans performed at 120 kV. A recent review reported that CT has higher diagnostic value for osteoporosis in Asian populations, particularly when non-enhanced CT is performed alone, participants are aged ≥ 65 years, and the proportion of women among the participants is high [38]. In this study, we enrolled Japanese patients undergoing hemodialysis who underwent non-enhanced CT; however, the patients were relatively young, and men outnumbered women. Patients undergoing hemodialysis represent a unique population characterized by profound disturbances in mineral metabolism, chronic inflammation, endocrine abnormalities, and accelerated bone loss [1,2]. Despite differences between our results and those of previous studies, the strength of the association between L1 CT-attenuation and BMD observed in our study was comparable to that reported in general population cohorts. Additionally, the L1 CT-attenuation cutoffs for diagnostic performance and clinical interpretation in our study were comparable to those for the general population cohorts. Our findings may validate L1 CT-attenuation as a robust surrogate marker for BMD and a screening tool for osteoporosis in patients on hemodialysis. Furthermore, the excellent intra- and inter-rater reproducibility and agreement observed in this study support the feasibility of incorporating this measurement into routine clinical practice, potentially through automated, software-based analysis with artificial intelligence in the future [39,40].

In the present study, L1 CT-attenuation was significantly associated with osteoporosis. Importantly, analyses using restricted cubic splines showed no evidence of non-linearity, indicating that this association can be adequately described by a linear model. These findings support the validity of linear regression analyses and suggest that L1 CT-attenuation provides a consistent estimate of bone fragility risk across its observed range.

The present study has some limitations. First, it was a retrospective single-center study with only a small number of Japanese participants, which may limit the generalizability of the findings. In addition, our study population consisted of patients who underwent routine annual checkups, which may represent a relatively healthier subset of the hemodialysis population. This could introduce selection bias and also limit the generalizability of our findings to patients who do not undergo regular checkups. Second, because of the cross-sectional design, causal relationships cannot be established, and the possibility of reverse causation cannot be ruled out. Third, the lack of internal or external validation, such as split-sample validation or an independent cohort, limits the external validity of our findings. Fourth, we cross-sectionally assessed L1 CT-attenuation and BMDs of femoral neck and lumbar spine at baseline; therefore, future fracture incidence and longitudinal changes in CT attenuation were not evaluated. Fifth, although L1 CT-attenuation provides valuable information on bone density, it cannot replace the comprehensive assessment of bone turnover or histological evaluation, which may be particularly relevant in CKD-related bone diseases. Future prospective multicenter studies with larger, ethnically diverse cohorts are needed to validate these thresholds across different CT platforms; moreover, in these studies, the ability of L1 CT-attenuation to predict incident fractures can be examined in patients undergoing hemodialysis. Furthermore, automated CT-based bone assessment should be integrated into radiological workflows, which may further enhance the early detection of osteoporosis and improve patient outcomes.

In conclusion, L1 CT-attenuation was independently associated with BMD of the femoral neck and lumbar spine and may be a useful screening tool for osteoporosis in Japanese patients undergoing hemodialysis. Opportunistic evaluation of L1 CT-attenuation using abdominal CT scans is a valuable and cost-effective approach for identifying patients at a high risk of osteoporosis, thereby facilitating early intervention, and potentially reducing fracture-related morbidity in this vulnerable population.

## Data Availability

The data that support the findings of this study are not publicly available due to privacy reasons but are available from the corresponding author upon reasonable request.
